# Orienting patients to greater opioid safety: models of community pharmacy-based naloxone

**DOI:** 10.1186/s12954-015-0058-x

**Published:** 2015-08-06

**Authors:** Traci C. Green, Emily F Dauria, Jeffrey Bratberg, Corey S. Davis, Alexander Y Walley

**Affiliations:** Department of Emergency Medicine, Rhode Island Hospital, Injury Prevention Center, 55 Claverick St., 2nd Floor, Providence, Rhode Island 02903 USA; Boston Medical Center, Injury Prevention Center, Boston University School of Medicine, 771 Albany St., Boston, Massachusetts 02118 USA; The Warren Alpert Medical School at Brown University, 222 Richmond St, Providence, Rhode Island 02903 USA; College of Pharmacy, University of Rhode Island, 7 Greenhouse Rd, Kingston, Rhode Island USA; Network for Public Health Law, Carrboro, North Carolina USA; Clinical Addiction Research Education Unit, Boston University School of Medicine/ Boston Medical Center, 801 Massachusetts Avenue, 2nd Floor, Boston, Massachusetts 02118 USA; 771 Albany St., Boston, Massachusetts 02118 USA

## Abstract

The leading cause of adult injury death in the USA is drug overdose, the majority of which involves prescription opioid medications. Outside of the USA, deaths by drug overdose are also on the rise, and overdose is a leading cause of death for drug users. Reducing overdose risk while maintaining access to prescription opioids when medically indicated requires careful consideration of how opioids are prescribed and dispensed, how patients use them, how they interact with other medications, and how they are safely stored. Pharmacists, highly trained professionals expert at detecting and managing medication errors and drug-drug interactions, safe dispensing, and patient counseling, are an under-utilized asset in addressing overdose in the US and globally. Pharmacies provide a high-yield setting where patient and caregiver customers can access naloxone—an opioid antagonist that reverses opioid overdose—and overdose prevention counseling. This case study briefly describes and provides two US state-specific examples of innovative policy models of pharmacy-based naloxone, implemented to reduce overdose events and improve opioid safety: Collaborative Pharmacy Practice Agreements and Pharmacy Standing Orders.

## Background

For thousands of years, pharmacies have served as a source of preventative and palliative medications—including antidotes to poisons—with pharmacists as the compounder and educator for these preparations [1]. Pharmacists are consistently ranked as one of the most trusted professions by consumers, viewed as both honest and ethical [2]. This perception is likely partly a result of their principled and important contributions to public health, such as offering vaccinations, selling sterile syringes, stocking emergency contraception, and refusing to sell tobacco products. Based on their specialized training and the role community pharmacies play in the United States (US) and global health systems, pharmacists are particularly well positioned to increase opioid safety, counsel patients, caregivers, and customers about overdose risk reduction, and provide naloxone rescue kits to the community. Despite these strengths, pharmacists are under-utilized within efforts to address the opioid crisis currently affecting the US.

Life expectancy in the US is among the shortest of all high-income countries, driven by high rates of mortality in people under 50 years of age, who are dying at unprecedented rates of unintentional drug overdose [3]. Drug overdose deaths have increased nearly sixfold since 1980, making it the leading cause of adult injury death in the US, surpassing deaths from motor vehicle crashes [4]. Opioids are the most common drugs involved in overdose deaths, with the majority being prescription opioid medications [5]. The US Centers for Disease Control and Prevention (CDC) have declared the current conditions an “epidemic” of overdose [6]. Outside of the US, overdose mortality trends in the European Union countries show persistently high and, in some countries, increasing rates of fatal overdose [7]. The World Health Organization reports that overdose is a leading cause of death among people who inject drugs [8]. Opioids are a cornerstone of modern medicine, providing critical relief for individuals suffering acute and some chronic pain conditions, thus full restriction of these drugs is impossible, impractical, and inhumane. To live more safely with access to opioid medications requires a more careful consideration of mechanisms for medical systems to more safely furnish opioids and for patients to use them while protecting themselves and their families from adverse events like over-sedation and overdose.

Medical experts first called for the provision of naloxone outside of the medical setting in the early 1990s [9]. By the mid-1990s, naloxone was being distributed to heroin users in Italy [10], Germany, and the UK [11,12]. The first programs to dispense naloxone in the US to people who use drugs began in the late 1990s and early 2000s, beginning in Chicago [13], then San Francisco [14]. By the mid 2000s, community-based programs in several US states (e.g., New Mexico, Massachusetts (MA), and New York) had begun distributing naloxone and overdose prevention and response training to people who use drugs and bystanders likely to witness an overdose [15]. As of June 2014, 644 community-based overdose education and naloxone distribution (OEND) programs were in operation in the US, and participants reported reversing more than 26,463 overdose events [16]. With 152,283 doses of naloxone dispensed since 1996, community-based programs were the cornerstone of OEND. As occurred during the 1990s with syringe access in the US [17], this paper considers models for expanding naloxone as one of several harm reduction supplies provided in the pharmacy setting.

The US pharmacy possesses several unique traits. US pharmacies are often found in highly visible, highly accessible locations, as both freestanding independently owned or chain drug stores, and integrated into department stores and supermarkets. Many offer the convenience of extended hours of pharmacy and store operations. Different from pharmacies in other countries [18], US pharmacies often sell an extensive range of retail items beyond prescription and over the counter medications, from milk and snacks to stationery, household goods, and seasonal items. Motivating this iteration of the modern US pharmacy are a focus on accessibility of commonly used household and personal products for purchase (whether visiting a pharmacy for a prescription or not) and the convenient placement of non-prescription items for purchase while awaiting a prescription to be filled. This environment serves people across varying socioeconomic strata, as one report showed that the equivalent of the entire US population visits pharmacies each week [19]. With respect to treatments for substance use disorders, although buprenorphine-based medications are dispensed there, traditionally, modern US pharmacies do not dispense methadone or heroin, do not observe dosing of therapies, and do not administer medications (except for vaccines). In contrast, many community pharmacies in Europe and Australia are woven into the fabric of harm reduction and medical services, and have fewer limits on medication dispensing paradigms [20,21]. Naloxone appears to be an exception to this supply pattern, as naloxone access is generally limited to health professionals, and in many countries [22] and is of limited availability in medical settings such as emergency medical service providers [23]. Uniquely, Scotland has made naloxone available in pharmacies without prescription [24], and Australia is considering a similar status (i.e., pharmacist only medication). For this paper, we focus on the role that pharmacies and pharmacists can potentially play in expanding access to the overdose antidote naloxone in the US.

Optimizing the pharmacist’s accessible, pivotal, and trusted position in the community is an under-utilized strategy to promote broader public health goals and reduce health disparities [25]. In the context of overdose prevention, a key component of this strategy is to more directly involve pharmacists in the provision of overdose prevention information and services, including expanding provision of the medication naloxone.

Pharmacists can detect medication prescribing errors and unintended interactions, and regularly interface with system tools designed to detect inappropriate prescribing such as prescription-monitoring programs (PMPs), which, in the US, are state-run electronic databases containing records of medications with abuse potential that are prescribed to the patient. Unfortunately, in many cases, the actions available to pharmacists, when a PMP query indicates a possible heightened risk of overdose, are limited [26]. Our previous qualitative work with pharmacists and people who inject drugs in Rhode Island (RI) suggests the following potential strategy for pharmacists to reduce fatal overdose risk: pharmacy-based naloxone (PBN) distribution [27]. We found that pharmacy-based interventions are feasible, desirable, and accessible to people who are at risk for overdose [27,28]. Pharmacists expressed interest in overdose prevention interventions, especially those that provide an opportunity to promote safe opioid use among illicit users, reduce risks of nonmedical opioid use for patients, respect the time constraints and limited space in most pharmacies, and capitalize on pharmacist’s professional and patient-oriented skills. A recent study of pharmacists in San Francisco concurred [29].

Naloxone, a prescription medication, reverses opioid-induced respiratory depression (the severe forms of which are termed “overdose”). It takes effect within minutes with a half life of 45–70 min, although these values differ with route of administration, the opioid consumed, and patient characteristics [30]. Naloxone is not a controlled substance (i.e., it has no potential for abuse and thus is not scheduled under US or international law) and has been used by medical personnel in the US for more than 40 years as the standard treatment for opioid overdose [31]. During the past decade, a number of initiatives have been undertaken to expand community access to naloxone, mainly by distributing naloxone rescue kits to people at an increased risk of overdose as well as members of their social network [32]. Kits typically contain two doses of the medication, instructions on use, and often, other safety materials such as a face shield for rescue breathing, alcohol swabs, and rubber gloves. These efforts were initially concentrated within needle and syringe access programs and other harm reduction service providers, and although they are increasingly supported by public health agencies and community-based organizations, they frequently operate outside of mainstream healthcare systems [15,16]. While initial reviews of these programs are positive [33–35], access to them is inadequate to reach people at risk of overdose who do not live in locations with harm reduction services, or who may be uncomfortable seeking these services.

The past five years have seen a dramatic upswing in efforts to expand access to naloxone in the US [16]. Increased naloxone access is now championed by a large number of mainstream organizations including the American Medical Association [36], the Office of National Drug Control Policy [37], and the World Health Organization [22]. One of the largest barriers to expanded naloxone access is the medication’s prescription status, which can only be changed by the Food and Drug Administration (FDA). Consequently, many of the recent efforts have involved changes to state law to permit naloxone to be distributed outside of the traditional prescriber-patient relationship. To date, over half of the US and the District of Columbia have modified state law to increase access to naloxone [38].

These legislative changes all have the goal of increasing naloxone access; however, they vary somewhat by state. As of June 2015, 32 states have passed laws permitting prescribers to write naloxone prescriptions for people other than the person at risk of overdose (i.e., “carers”, or friends and family members of those at high overdose risk). Additionally, 21 states have passed legislation allowing “standing” or non-patient-specific naloxone prescriptions, which permit naloxone dispensing to anyone who meets specified criteria (e.g., at risk of experiencing or witnessing overdose). Nearly all of these laws provide civil immunity for the prescriber, and 26 extend limited immunity from criminal prosecution for a person who reports an overdose in good faith [39]. Numerous states have also taken steps to equip police officers and other first responders with naloxone [40,41].

More recently, legal changes focus on mechanisms to directly involve pharmacies and pharmacists in broader naloxone access. While moving naloxone to over the counter status might improve access to the medication, the FDA has shown little interest in pursuing that path to date [42]. Instead, states have innovated Collaborative Pharmacy Practice Agreements, Pharmacy Standing Orders, Naloxone Provision Per Protocol, and Pharmacist as Prescriber mechanisms, which effectively place naloxone “behind the pharmacy counter”. To put PBN in context, Table 1 overviews the naloxone distribution models observed in the US to date. Models include OEND via community-based organizations [16], via traditional prescription (from a prescriber-pharmacist dyad), and via PBN. US communities may employ one or more models to augment naloxone distribution, depending on the local epidemiology of opioid overdose, political will, and legal barriers, among other factors. Based on our experience as the first state-wide PBN program, we briefly describe and provide state-specific examples of the following two innovative policy models of PBN, implemented to reduce overdose events and improve opioid safety: Collaborative Pharmacy Practice Agreements and Pharmacy Standing Orders.

**Table 1:** Naloxone distribution models in the USA: prescription specifications, targeted at-risk populations, and geographic reach

	Community-based organization Naloxone distribution	Traditional prescription	Pharmacy-based Naloxone models			
			CPA	Standing medication order	Protocol order	Pharmacist prescribing
Who issues prescription?	Prescriber via standing order	Prescriber	Non-pharmacist prescriber	Non-pharmacist prescriber	Licensing board	Pharmacist
Medical professionals required	Varies by state: prescriber, state/local health department	Prescriber + pharmacist	Prescriber + pharmacist	Prescriber + pharmacist	Pharmacist	Pharmacist
Potential recipients	Individuals served by the community-based organization	Patients of the prescriber	Varies by state	Anyone meeting criteria specified by prescriber	Anyone meeting criteria specified by licensing board	Anyone for whom medication is indicated
Target overdose risk population served	People who use drugs (prescription opioids, heroin) who access the community-based organization*	People who use drugs who are in treatment/visit a prescriber*	People who use drugs (prescription opioids, heroin) who visit a pharmacy*	People who use drugs (prescription opioids, heroin) who visit a pharmacy*	People who use drugs (prescription opioids, heroin) who visit a pharmacy*	People who use drugs (prescription opioids, heroin) who visit a pharmacy*
		Patients prescribed opioids who are at risk of overdose*	Patients filling a prescription for opioids at a pharmacy who are at risk of overdose*	Patients filling a prescription for opioids at a pharmacy who are at risk of overdose*	Patients filling a prescription for opioids at a pharmacy who are at risk of overdose*	Patients filling a prescription for opioids at a pharmacy who are at risk of overdose*
Geographic reach	Limited to where community-based organizations are located and operate	Limited to where the prescriber practices	Any participating pharmacy within the state	Any participating pharmacy within the state	Any participating pharmacy within the state	Limited to where the pharmacist practices

*A majority of states now permit prescriptions to be written for third parties (e.g., friends, staff of organizations that provide services to individuals at risk of overdose) as well as the person at risk of overdose

*CPA* collaborative practice agreement

## Case description

### Rhode Island: collaborative pharmacy practice agreement for naloxone

As part of a growing trend in pharmacy practice, collaborative pharmacy practice agreements (CPAs) have been implemented for a range of health conditions with public health impact, including vaccinations, Lyme disease prophylaxis, and diabetes management [43]. CPAs permit a pharmacist to work in collaboration with a prescriber for the purpose of drug therapy management of patients, pursuant to an authorized and agreed upon protocol. According to the American Pharmacists Association (APhA), 48 states permit pharmacists to enter into CPAs with prescribers to manage patient pharmaceutical care, and at least 21 states permit pharmacists to initiate medication therapy under such agreements [44]. These agreements can generally be used to provide naloxone to individuals who may be able to use it to save lives, even without legislative change.

Driving the involvement of pharmacists in the RI overdose epidemic was a clear public health need; since 2005, drug overdose deaths in the state outnumbered deaths from motor vehicle crashes, falls, firearms, and fire among adults under 85 years of age [45]. The vast majority of these overdose deaths involve prescription opioid medications. RI has the nation’s highest rate of illicit drug use per capita (15.6 % past month use, among those 12 and older, versus 8.9 % nationally) [46], and nonmedical use of prescription opioids ranks above the national average (5.18 % past month use, among those 12 and older, versus 4.57 % nationally) [47,48]. To address these stark statistics, the RI Board of Pharmacy approved a CPA for naloxone (CPAN), which began in 2011, initially as a pilot in five Walgreens Pharmacies in locations with high prescription opioid overdose mortality. The CPAN expanded to all Walgreens Pharmacies and any other interested pharmacies (e.g., CVSHealth), in response to an outbreak of synthetic fentanyl overdose deaths in Spring 2013, and was formalized in regulation in 2014 [49,50]. The Office of National Drug Control Policy has recognized the RI CPAN as a leading public health-commercial collaboration, and an important distribution model for naloxone to address overdose risk in the community [51]. When signed by any number of participating pharmacists and a single prescriber, the CPAN facilitates pharmacist-initiated prescription and provision of naloxone to eligible patients (Table 2).

**Table 2:** Eligibility criteria for patient participation in the Rhode Island collaborative practice agreement for naloxone (CPAN)

▪ Voluntarily request
▪ Recipient of emergency medical care for acute opioid poisoning
▪ Suspected illicit or nonmedical opioid user
▪ High dose opioid prescription (>100 morphine mg equivalents daily)
▪ Methadone prescription to opioid naïve patient
▪ Dispensed an opioid prescription and:
▪ History of smoking
▪ COPD
▪ Respiratory illness or obstruction
▪ Renal dysfunction or hepatic disease
▪ Known or suspected concurrent alcohol abuse
▪ Concurrent benzodiazepine prescription
▪ Concurrent SSRI or TCA anti-depressant prescription
▪ Recently released prisoners from a correctional facility
▪ Released from opioid detoxification or mandatory abstinence program
▪ Patients entering a methadone maintenance treatment program
▪ Patients that may have difficulty accessing emergency medical services

SOURCE: Rhode Island Board of Pharmacy, 2011

**Figure 1:**
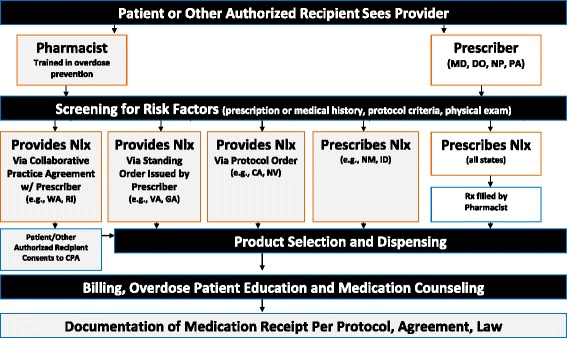
Pharmacy Naloxone Access Models. Process flow as experienced by patient and pharmacist. *Nlx* Naloxone, *Rx* prescription, *MD* medical doctor, *DO* doctor of osteopathic medicine, *NP* nurse practitioner, *PA* physician assistant, *CPA* collaborative practice agreement, State abbreviations: *WA* Washington, *RI* Rhode Island, *VA* Virginia, *GA* Georgia, *CA* California, *NV* Nevada, *NM* New Mexico, *ID* Idaho

**Figure 2:**
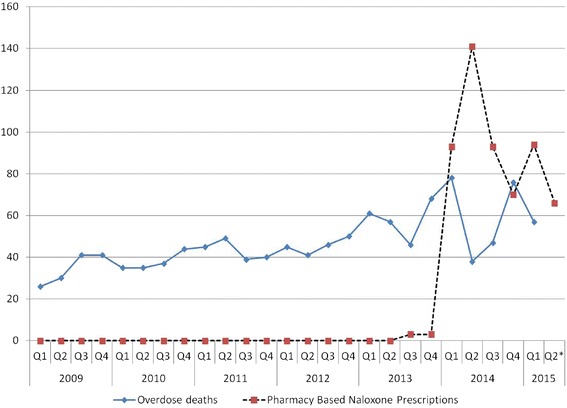
Overdose deaths and pharmacy-based naloxone prescriptions dispensed in Rhode Island, 2009 to 2015, by quarter. SOURCE: Rhode Island Department of Health, 2015

Per Pharmacy Board regulations, participating patients must consent for the exchange of information between pharmacist and prescriber, complete a brief enrollment form, and receive a handout with overdose education and medication specifications, then check-in with the pharmacist, who verifies their understanding of naloxone use before it is dispensed. Figure 1 depicts the process and interactions that broadly define provider-based naloxone access (i.e., outside of the community-based organization setting), beginning with when a patient approaches a provider for the medication, to naloxone receipt, and finally to documentation of medication provision. Involvement in the CPAN requires one hour of continuing professional education annually to sustain participation in the agreement, such as the “Prescribe to Prevent” online training for prescribers and pharmacists at opioidprescribing.com. The University of Rhode Island (URI) also developed an online continuing education program for pharmacists to receive the standard 1-h PBN training required to initiate their participation. As of June 2015, 363 pharmacists had completed the URI training, 96 % of whom were employed by retail chain pharmacies. During 2014, through the CPAN, RI roughly doubled the community-based distribution of naloxone. From January 2014 to May 2015, 572 PBN prescriptions were dispensed (Figure 2), comprising 25 % of all naloxone distributed in RI. While health departments of many neighboring states are reporting increases in overdose deaths from 2013 onward (New Hampshire, 2013 = 193 deaths, 2014 = 321 deaths [52]; Connecticut, 2013 = 490 deaths, 2014 = 558 deaths [53]; Massachusetts, 2013 = 967 deaths, 2014 = 1008 deaths projected [54]), RI experienced only seven more deaths in 2014 (*n* = 239) than in 2013 (*n* = 232) [55]. Other interventions to reduce overdose in RI were introduced contemporaneously (i.e., equipping law enforcement with naloxone, emergency department-based naloxone distribution), so causal attribution of the attenuation to PBN alone is not possible. Efforts to further expand PBN access will be evaluated beyond this proximal impact.

### Massachusetts: pharmacy standing order

More than 8926 people died of opioid overdoses in MA between 2000 and 2014 [54], mirroring national trends of increasing opioid overdose deaths. Different from most other states, however, MA has a well-established, community naloxone distribution network [35] that has operated since 2006. Under this model, the medical director (AYW) serves as the single prescriber to all community-based naloxone recipients. While this community network remains essential, it is not able to meet the growing need for naloxone. In March 2014, the Governor declared a public health emergency, and, in response, the MA Board of Registration in the Pharmacy and the Drug Control Program adopted regulations one month later that authorized pharmacists to dispense naloxone under a standing order signed by a physician [56]. In July 2014, a law went into effect that essentially mirrored the existing Order [57]. These steps were necessary in MA to accomplish broader “behind the pharmacy counter” access to naloxone, because existing CPA regulations only permit pharmacists to dispense medication to individuals that are established patients of the prescriber.

Pharmacy standing orders are similar to those in existence in many states for expedited partner treatment of sexually transmitted infections, which allows a pharmacist to dispense antibiotics to the partner of a person with a sexually transmitted infection without examining the partner. Figure 1 depicts the patient and pharmacist flow of interactions under a pharmacy standing order. In this way, PBN offers additional outlets, with immense geographic reach, to locales that may have less community naloxone distribution coverage and/or with elevated mortality risk.

The pharmacy standing order is conceptually similar to the CPA in terms of implementation and intended reach (Table 1), and that of MA includes many elements of the RI CPAN; the standing order must be signed by a physician and the pharmacy manager at each outlet; copies must be filed with the Board of Registration in Pharmacy and maintained onsite at the pharmacy; pharmacists need to have an understanding of the appropriate function and use of naloxone; counseling of the customer by the pharmacist; inclusion of an overdose prevention information sheet within the naloxone prescription; and labeling of the naloxone prescription with an expiration date. Who is eligible to receive naloxone under pharmacy standing orders is left up to the physician and pharmacist to determine in the agreement (Table 1). An August 2012 law explicitly permits prescribing both to people at risk of opioid overdose and people likely to be opioid overdose bystanders, thus the patient eligibility specified on the pharmacy standing orders typically includes both of these groups [58,59]. Between March and December 2014, 145 individual retail pharmacies within MA, primarily in communities with high rates of overdose mortality, filed a naloxone pharmacy standing order with the Board of Registration in Pharmacy. It will be some time before PBN meets or exceeds the high distribution volume of the community-based programs, but collaborations such as those between drug treatment programs and community pharmacies in many of the pharmacy locations with standing orders suggest that there are multiple local implementation prospects in high-overdose burden communities.

## Discussion

Here, we briefly outlined two models of PBN and provided state-specific examples that are immediately implementable within existing regulatory frameworks of many US states, and may serve as models in international settings. Indeed, the CPAN and pharmacy standing order are conceptually similar to other legal frameworks, such as national patient group directions [60], which serve as a structure for wider scale PBN in the UK [61]. Pharmacy-based naloxone is one public health intervention that better leverages pharmacies’ capacity and pharmacists’ skills. By utilizing well-established health systems, where appointments are not required, services are provided at low or no-cost, and trusted health professionals are accessible, PBN expands the reach of naloxone to individuals beyond those currently being served by community-based and harm reduction organizations. While implementation is early and more comprehensive evaluations are in order, PBN is being dispensed to patients and caregivers.

An important element of the described PBN models is a clear path for prescription drug reimbursement on public and private payer formularies, in addition to a consultation fee, similar to that paid for immunization administration. Currently, most major public and private insurers cover naloxone formulations; however, this reimbursement does not include a pharmacist’s consultation with the patient. The mass vaccination campaign of the 2009 H1N1 influenza vaccine may serve as a useful model for developing policies and/or executive orders that require health insurance coverage to include PBN naloxone and administrative fees. In order to extend the reach of the campaign, the federal government covered the cost of the vaccine itself and required that any provider agreeing to administer the vaccination should not charge an administrative fee that exceeded the region Medicare or Medicaid rate [62]. This reimbursement coverage extended to vaccinations provided in traditional (e.g., doctor’s offices, health care facilities) and non-traditional settings (e.g., pharmacies), and served as incentive for US pharmacies to adopt immunization administration.

In addition to reimbursement challenges, several barriers may limit the wide scale implementation of PBN. These barriers include access to formulation and products; legal misconceptions about naloxone prescription, dispensing and use; the need for education and advocacy at the patient-, familial-, social network-, and health care provider-levels (e.g., pharmacists, physicians, pharmacy, and medical schools and associations); and ethical considerations (i.e., pharmacists refusing naloxone to certain patients), among others [27,63–65]. Many of these challenges are not unique to the pharmacy, but rather, are shared by naloxone distribution models across outpatient and community settings. Future evaluations of PBN should assess for the presence of these and new challenges, to understand their impact and to measure the contribution of PBN to mortality reduction.

The two state examples differed considerably in their base rates of community naloxone availability, permitting different potential contributions of PBN to the risk environment. In RI, the limited competing community sources for naloxone meant that PBN was central to state-wide distribution efforts with greater geographic reach, whereas in MA, the pharmacies with standing orders provide additional outlets in communities with existing harm reduction support infrastructure and high-overdose burden. Some states may prioritize reaching certain high-risk populations through PBN. While every person who receives an opioid prescription is a potential candidate for PBN, special attention could be directed to those known to be at increased risk of opioid overdose and adverse events (Table 2), in a pharmacist-initiated rather than a patient-initiated PBN approach. Strategies for improved implementation and for maximizing PBN’s reach are ripe areas of research.

Recently, APhA and the National Association of Boards of Pharmacy formally recognized the role of the pharmacist in overdose recognition, education, and furnishing naloxone [66,67] The evolving role of pharmacies in this area suggests a number of approaches beyond PBN for directly or indirectly addressing the opioid epidemic, including: 1) pharmacy-prescriber partnerships to better manage opioid medications, reduce overprescribing, and increase access to addiction care; 2) counseling about and supplying patients with safe storage tools [68,69]; and 3) increasing community access to treatment of opioid-use disorders with evidence-based medicines (i.e., in-pharmacy buprenorphine prescribing, and daily dispensing of methadone or buprenorphine in areas with inadequate access to licensed opioid treatment programs, and in-pharmacy naltrexone injections), as is common in many international settings [21,70].

## Conclusions

PBN, exemplified by state models described in RI and MA, can be adopted and applied elsewhere nationally and internationally as a feasible mechanism for expanded access to naloxone. PBN may provide a pivotal foundation and catalyst for pharmacy-based services from which pharmacists can have more central roles as facilitators and advocates for treatment and recovery. For example, in several countries including Australia, Scotland, Germany, and Canada [20,21,71,72], community pharmacies are a major point (and in some localities, the only point) [21] of effective treatment of opioid-use disorders; co-locating naloxone access alongside such provision is consistent with pharmacy harm reduction service delivery, and generally supportive attitudes of pharmacists toward harm reduction [70], as several communities in Scotland have accomplished [73]. With an unprecedented number of opioid overdoses globally [22], an expanding pool of opioid use initiates in the US [74], and an aging baby boomer generation with high lifetime drug use and high burdens of chronic pain often treated with opioids [75,76], it is imperative that creative, sustainable solutions, such as PBN, are implemented.

## Abbreviations

APhA: American Pharmacists Association
CPA: collaborative pharmacy practice agreement
CPAN: collaborative pharmacy practice agreement for naloxone
FDA: United States Food and Drug Administration
MA: Massachusetts
OEND: overdose education and naloxone distribution
PBN: pharmacy-based naloxone
PMP: prescription-monitoring program
RI: Rhode Island
SAMHSA: Substance Abuse and Mental Health Services Administration
URI: University of Rhode Island
US: United States of America
